# Body Acceleration as Indicator for Walking Economy in an Ageing Population

**DOI:** 10.1371/journal.pone.0141431

**Published:** 2015-10-29

**Authors:** Giulio Valenti, Alberto G. Bonomi, Klaas R. Westerterp

**Affiliations:** 1 Department of Human Biology, Maastricht University, Maastricht, The Netherlands; 2 Personal Health Solutions, Philips Research Laboratories, Eindhoven, The Netherlands; West Virginia University School of Medicine, UNITED STATES

## Abstract

**Background:**

In adults, walking economy declines with increasing age and negatively influences walking speed. This study aims at detecting determinants of walking economy from body acceleration during walking in an ageing population.

**Methods:**

35 healthy elderly (18 males, age 51 to 83 y, BMI 25.5±2.4 kg/m2) walked on a treadmill. Energy expenditure was measured with indirect calorimetry while body acceleration was sampled at 60Hz with a tri-axial accelerometer (GT3X+, ActiGraph), positioned on the lower back. Walking economy was measured as lowest energy needed to displace one kilogram of body mass for one meter while walking (WCost_min_, J/m/kg). Gait features were extracted from the acceleration signal and included in a model to predict WCost_min_.

**Results:**

On average WCost_min_ was 2.43±0.42 J/m/kg and correlated significantly with gait rate (r^2^ = 0.21, p<0.01) and regularity along the frontal (anteroposterior) and lateral (mediolateral) axes (r^2^ = 0.16, p<0.05 and r^2^ = 0.12, p<0.05 respectively). Together, the three variables explained 46% of the inter-subject variance (p<0.001) with a standard error of estimate of 0.30 J/m/kg. WCost_min_ and regularity along the frontal and lateral axes were related to age (WCost_min_: r^2^ = 0.44, p<0.001; regularity: r^2^ = 0.16, p<0.05 and r^2^ = 0.12, p<0.05 respectively frontal and lateral).

**Conclusions:**

The age associated decline in walking economy is induced by the adoption of an increased gait rate and by irregular body acceleration in the horizontal plane.

## Introduction

Walking is a periodic motor task that allows a body to move over a distance [1]. Characteristics of walking such as walking speed, gait rate and regularity change with age: elderly show reduced walking speed and regularity together with increased gait rate [2]. These changes result from declining fitness and decreased walking stability and are associated with fall risk [3, 4]. Results of decreased walking speed and increased fall risk include functional decline with a reduction of life expectancy of up to 30% between similar aged subjects with different walking speeds [5–11].

Together with reduced walking speed, ageing is associated with low walking economy, i.e. increased walking cost [12]. Walking cost is quantified as the energy required to displace 1 kg of body mass over 1 meter and follows a u-shaped relation with walking speed, showing a minimum between 0.9 m/s and 1.4 m/s [13–16]. The walking speed at which the minimum is measured has been defined as optimal walking speed and approximates subjects’ preferred walking speed [17, 18]. A gradual increase of minimum walking costs begins after the 50^th^ year of age and progressively speeds up after the age of 65 y possibly leading to reduced mobility in elderly [15, 19, 20]. It has been suggested that increased walking cost together with diminished energetic capacity could contribute to the decline of walking speed [19, 21].

The walking cost is minimized by reducing muscle activation through precise sequencing of muscle activation and coordination of acceleration and deceleration of body segments [22]. The result of this strategy is a smoother velocity profile of body segments with reduced and more regular acceleration of the center of body mass [23, 24]. Ageing affects the coordination and the strength of muscles and therefore body accelerations and walking economy might be related in elderly. In particular, the age-related reduction in walking economy could be reflected in irregular acceleration of the center of body mass, and result in reduced walking speed.

Wearable sensors such as accelerometers are able to collect continuous data and have been used to measure cycle by cycle variations in gait rate and regularity of gait cycles [25], showing that decreased regularity of acceleration patterns is associated with aging and fall risk, functional decline and eventually mortality [26–30]. The trunk and limbs play a critical role in the regulation body acceleration during walking [31]. Symmetrical walks can be modeled as an inverted pendulum where the body mass is concentrated in a virtual point called center of mass. This model describes the cinematic strategy to preserve mechanical energy by periodically transforming kinetic energy into potential energy and vice versa. The lower back is the closest position to the center of body mass. Asymmetrical walks deviate from this model and changes in limbs movements have to compensate for the deviations and maintain stability. This results in increased energy expenditure and decreased walking economy in asymmetrical walks. Nevertheless, healthy subject show symmetrical walking patterns and therefore their walking patterns can be described by the inverted pendulum model and by the accelerations of the center of body mass. Although an interaction between trunk acceleration and walking economy has been hypothesized, simultaneous measurements of energy expenditure and body acceleration during walking have not been reported yet [32, 33].

The current study aims at detecting determinants of walking economy from body acceleration during walking in an ageing population in order to define innovative indicators for functional performance and fall risk.

## Methods

### Population

Thirty-five healthy subjects (18 men and 17 women) aged 51 to 83, 64±8 years on average were recruited by advertisements in local newspapers (Table 1). After signing a written informed consent, respondents completed a questionnaire including orthopedic conditions, neurological disorders and cardiovascular problems that could affect the study. The questionnaire was discussed during a medical visit with a doctor. All subjects were in good orthopedic, neurological and cardiovascular conditions and were therefore included. The study was conducted according to the Declaration of Helsinki and the Ethics Committee of the Maastricht University Medical Center approved the study. This trial was registered at www.clinicaltrials.gov as NCT01609764.

**Table 1:** Subject Characteristics (Mean±SD).

Gender (m/f)		18/17
Age (y)		64±8
Height (m)		1.70±0.10
Body mass (kg)		73.8±11.7
BMI (kg/m^2^)		25.5±2.4
Walking economy (J/m/kg)		2.43±0.42
Step Length (m)		0.65±0.9
Gait Rate (Hz)		0.91±0.09
Regularity (%)	Vertical	29±7
	Lateral	51±13
	Frontal	43 ±12

BMI, body mass index; Walking economy, the lowest energy needed to displace one kilogram of body mass for one meter while walking; Regularity, deviations of body accelerations from a personalized template as a percentage of the variance of the template. Lower values indicate higher regularity

### Study design

The testing protocol included standing and walking on a treadmill at selected speeds encompassing the preferred speed. Four walking speeds at regular intervals were chosen out of six: 0.28 m/s, 0.56 m/s, 0.83 m/s, 1.11 m/s, 1.39 m/s and 1.67 m/s. The protocol started at 0.83 m/s and was increased every 5 minutes by 0.28 m/s. If the subject was able to walk all 4 speeds up to 1.67 m/s without running, the test ended. Instead, if the walking speed exceeded subject’s running threshold, the test was continued at the lower speeds to obtain 4 speeds at regular intervals for each subject. Subjects wore a mask to measure energy expenditure and an accelerometer on the lower back to measure regularity of acceleration patterns of gait and step rate. Tests were performed in the morning, when subjects were fasted and had not undergone intense physical activity.

### Energy expenditure

During the protocol, oxygen consumption and carbon dioxide production were measured with an indirect calorimeter (Omnical, Maastricht Instruments, Maastricht, The Netherlands). Calculation of total energy expenditure from O2 consumption and CO2 production was based on Brouwer’s formula [34].

For each subject, total energy expenditure was averaged over the plateau during the last 2 minutes of standing and of each walking speed. The energy expenditure of standing was subtracted from total energy expenditure to calculate walking energy expenditure. Walking cost was defined as the energy needed to displace one kilogram of body mass for one meter while walking, and it was calculated as walking energy expenditure normalized for walking speed and for body mass. The minimum walking cost (WCost_min_) was considered for further analysis and the speed at which it was measured was defined as optimal walking speed.

### Body acceleration

During the protocol, body accelerations were sampled at 60 Hz, with a GT3X++ (ActiGraph, Pensacola, FL) positioned on the lower back using a belt. Tri-axial body acceleration data were downloaded and exported to Matlab (Mathworks, Natick, MA). The reference axes of the data (x, y, z) were aligned to the anatomical axes (vertical, lateral, frontal). Only the data recorded during the last 2 minutes of the optimal walking speed was considered for further analysis. Gait rate was calculated as the peak in the power spectrum density of the vertical axis, calculated between 0.5 and 1.5 Hz.

### Regularity of acceleration patterns of gait

**Fig 1:**
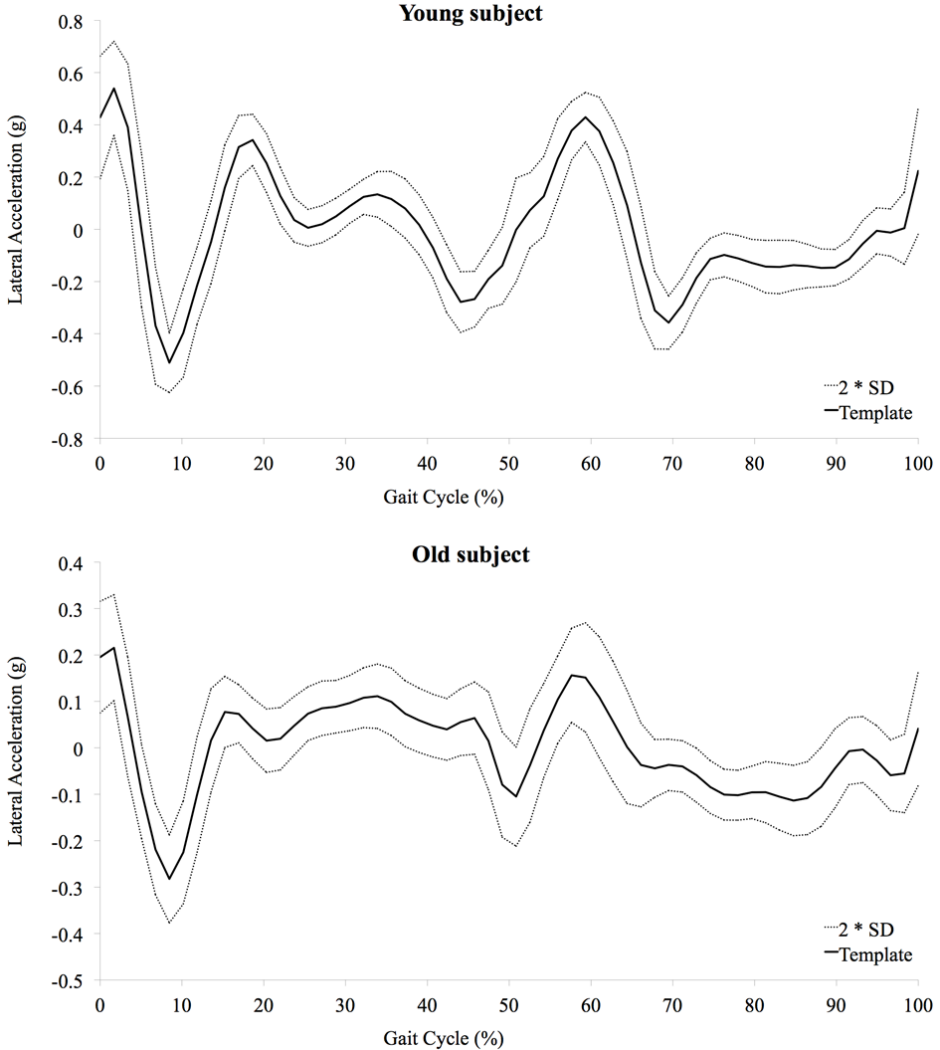
Example of two acceleration templates in the lateral direction with twice the standard deviation of all steps from the template. The first example refers to a younger subject (55y old) while the second refers to an older one (83y old). Regularity was quantified as the Standard Deviation From the Template, Expressed as a Percentage of the Standard Deviation of the Template. Lower values indicate higher regularity. SD, standard deviation of all steps from the template; Template, average of all steps.

Regularity of acceleration patterns of gait was quantified as deviation of the acceleration from a personalized template. For each subject, acceleration peaks in the vertical direction were identified at approximately twice the gait rate and used to detect foot contact with the ground. Acceleration of each gate cycle included samples between two consecutive contacts of the same foot. Gait cycles were re-sampled to the slowest gait cycle allowing a uniform number of samples and avoiding aliasing. After aligning all gait cycles, means and standard deviations of corresponding samples were calculated. The sequence of the means formed a personalized template of the acceleration patterns of gait (Fig 1). The standard deviation of the means was a measure of the power of the template, while the average of the standard deviations was a measure of deviation of all gait cycles from the template [35]. Regularity was calculated as the ratio between the deviation of all gait cycles from the template and the power of the template, expressed as a percentage. Lower percentage indicates higher regularity and vice versa.

### Statistical analysis

Stepwise multiple-linear regression analysis was used to include the best independent variables including gait rate and regularity in the three directions to predict WCost_min_. Squared Pearson product-moment correlation coefficient (r^2^) was used to describe associations between variables. Data elaboration was conducted in MATLAB ®. Regression analysis was conducted in SPSS®. All variables are expressed as mean ± standard deviation. The significance threshold was set to p<0.05.

## Results

The optimal walking speed, i.e. the walking speed with the lowest walking cost, was on average 1.18±0.16 m/s. No significant difference between male and female (18 males vs 17 females) was found in age, optimal speed or WCost_min_, therefore they were analyzed together.

**Fig 2:**
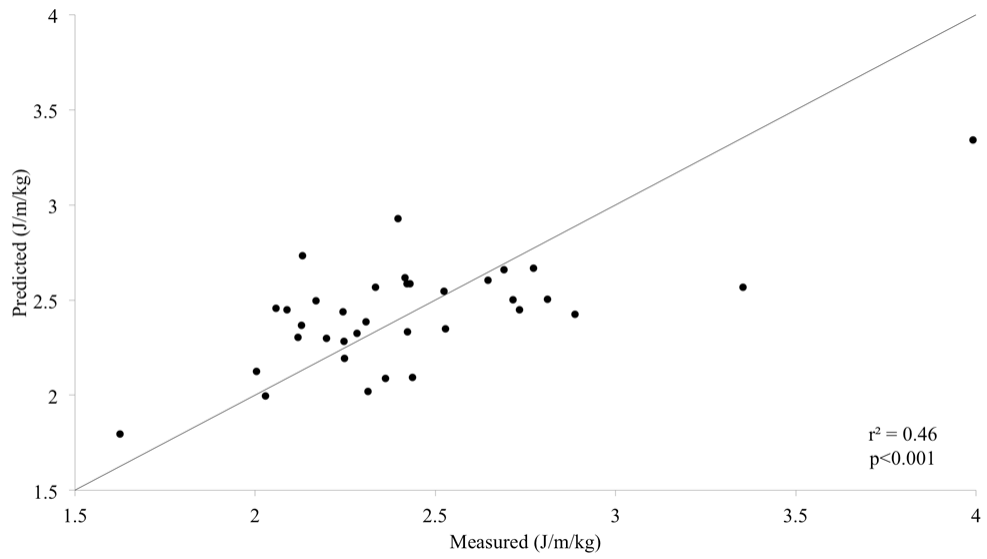
Predicted minimum walking cost as a function of measured values with the line of identity. r^2^, squared correlation between estimates and measures.

On average WCost_min_ was 2.43±0.42 J/m/kg and correlated significantly with gait rate (r^2^ = 0.21, p<0.01) and regularity along the frontal and lateral axes (r^2^ = 0.16, p<0.05 and r^2^ = 0.12, p<0.05 respectively). Together, the three variables explained 46% of the inter-subject variance (p<0.001, Fig 2) with a standard error of estimate of 0.30 J/m/kg, which was 13% of the average WCost_min_. The model is described in Table 2; variables are presented in order of inclusion.

**Table 2:** Stepwise Regression of the Minimum Walking Cost, with an Accelerometer in Elderly. Variables are presented in Order of Inclusion.

Variable	Coefficient	Significance	r^2^	SEE (J/m/kg)
Intercept	-0.699	-		
Gait Rate	2.300	p<0.005		
Regularity Lateral	1.254	p<0.01		
Regularity Frontal	0.941	p<0.05		
Model			0.46	0.30

Variable, variables included in the stepwise regression; r^2^, squared correlation between estimates and measures; SEE, standard estimation error; Regularity Lateral, deviations of body accelerations from a personalized template in the lateral direction. Lower values indicate higher regularity; Regularity Frontal, deviations of body accelerations from a personalized template in the frontal direction. Lower values indicate higher regularity

A significant correlation was found between WCost_min_ and age (r^2^ = 0.44, p<0.001), and between estimated WCost_min_ and age (r^2^ = 0.32, p<0.01). Decreasing regularity along the frontal and lateral axes was positively related with age (r^2^ = 0.16, p<0.05 and r^2^ = 0.12, p<0.05 respectively).

## Discussion

The study showed that the age related decline in walking economy is associated with the adoption of an increased gait rate and by irregular body acceleration in the horizontal plane. Estimates of WCost_min_ from step rate and regularity in the horizontal plane could describe part of the covariance between walking economy and age.

Measures of WCost_min_ and gait rate (2.43±0.42 J/m/kg and 0.91±0.09 J/m/kg respectively; mean±SD) were in line with the literature [36, 37]. Decreased walking economy, measured as increased WCost_min_, was positively related with gait rate (r^2^ = 0.21, p<0.01). A higher gait rate implies that more steps are made per unit of distance explaining the increase of WCost_min_. Higher gait rate is associated to shorter step length which characterizes a less destabilizing gait [38]. It is therefore concluded that a decreased walking economy is associated with a destabilizing gait.

A significant relation was found between WCost_min_ and regularity along the frontal and lateral axes (r^2^ = 0.16, p<0.05 and r^2^ = 0.12, p<0.05 respectively) while regularity in the vertical direction didn’t influence WCost_min_. Decreased regularity in the horizontal plane may result from reduced control of the movements, which may lead to instability in the horizontal plane. Possible causes are increased stiffness of the articulations and loss in muscle mass which have been reported in elderly [36]. The reduced regularity in the horizontal plane could have induced elderly to increase gait rate with a consequent rise in WCost_min_.

A step-wise multilinear model, including step rate and regularity in the horizontal plane, could estimate WCost_min_ and could describe part of the covariance between walking economy and age (WCost_min_ and age: r^2^ = 0.44, p<0.001; WCost_min_ estimates and age r^2^ = 0.32, p<0.01). Step rate and regularity in the horizontal plane could therefore partly determine the age-related increase in WCost_min_. The unexplained part of covariance instead could be due to the age related increase in coactivation of antagonist muscles, which would rise the cost of walking [36] without necessarily affecting the acceleration patterns of gait. Furthermore, coactivation of antagonist muscles has been shown to be a compensatory response to instable conditions [39] and can therefore be a consequence of the reduced regularity in the horizontal plane.

A limitation of this study is that muscle activity during walking was not measured. Measures of muscle activity could have described how ageing induces less stable and therefore less efficient walking in this population. Nevertheless the measurement of muscle activity requires electrodes placed on the lower limbs, which would alter one’s gait cycle and consequently affect the cost of walking. All the tests were conducted inside a laboratory to increase the accuracy of the results. The generalization of these results to daily life requires further studies in order to provide further insight in the ageing process, and to prevent and delay functional decline and increased fall risk associated with ageing.

Increased fall risk is one of the most common comorbidities related to ageing [40–42]. Reduced walking speed is an indicator of fall risk [5–9] and is related to reduced walking economy [19, 21]. Interest in fall risk assessment in daily life with unobtrusive accelerometers is increasing. Laboratory measures of acceleration during standing or during walking have been proposed to discriminate between fallers and non-fallers, although validation in daily life is required [43]. The results of this study suggest that changes in walking economy can be detected with an accelerometer. Given that walking economy is related to walking speed and therefore fall risk, these results might suggest that regularity in the horizontal plane could contribute to fall risk assessment. In this population fall risk was not evaluated, but further studies focusing on the capability of the described determinants to detect increased fall risk could allow the design of specific interventions to prevent subjects’ falls.

Future studies could aim at extending these results in daily life where they might contribute to the prediction of falling risk. The features described in this study could be measured in older adults with a history of falls and older adults with no history of falls. This would reveal a possible direct relation between the features and fall risk. In case this relation was confirmed, the features could be implemented in devices capable of detecting falls in free living [44–46]. Simultaneous measure of the features and of fall history would allow a quantification of the predictive power of the features for fall risk. Future studies could also define the mechanisms behind the effect of irregular body acceleration on walking economy by including measures of muscle activations. Possibly, wireless systems could reduce the obtrusiveness of most current electromiographic systems.

In conclusion, increased gait rate and by irregular body acceleration in the horizontal plane contribute to the age associated decline in walking economy. A waist-worn accelerometer can assess regularity of body acceleration and estimate walking economy, with possible applications in fall prevention and the delay of functional decline in the elderly.

## Abbreviations

WCost_min_: the lowest energy needed to displace one kilogram of body mass for one meter while walking
